# Design of the sex hormones and physical exercise (SHAPE) study

**DOI:** 10.1186/1471-2458-7-232

**Published:** 2007-09-04

**Authors:** Evelyn M Monninkhof, Petra HM Peeters, Albertine J Schuit

**Affiliations:** 1 Julius Center for Health Sciences and Primary Care, University Medical Center Utrecht, Utrecht, The Netherlands; 2 Division of Public Health and Health Care, National Institute for Public Health and the Environment, Bilthoven, The Netherlands

## Abstract

**Background:**

Physical activity has been associated with a decreased risk for breast cancer. The biological mechanismn(s) underlying the association between physical activity and breast cancer is not clear. Most prominent hypothesis is that physical activity may protect against breast cancer through reduced lifetime exposure to endogenous hormones either direct, or indirect by preventing overweight and abdominal adiposity. In order to get more insight in the causal pathway between physical activity and breast cancer risk, we designed the *Sex Hormones and Physical Exercise (SHAPE) *study. Purpose of SHAPE study is to examine the effects of a 1-year moderate-to-vigorous intensity exercise programme on endogenous hormone levels associated with breast cancer among sedentary postmenopausal women and whether the amount of total body fat or abdominal fat mediates the effects.

**Methods/Design:**

In the SHAPE study, 189 sedentary postmenopausal women, aged 50–69 years, are randomly allocated to an intervention or a control group. The intervention consists of an 1-year moderate-to-vigorous intensity aerobic and strenght training exercise programme. Partcipants allocated to the control group are requested to retain their habitual exercise pattern. Primary study parameters measured at baseline, at four months and at 12 months are: serum concentrations of endogenous estrogens, endogenous androgens, sex hormone binding globuline and insuline. Other study parameters include: amount of total and abdominal fat, weight, BMI, body fat distribution, physical fitness, blood pressure and lifestyle factors.

**Discussion:**

This study will contribute to the body of evidence relating physical activity and breast cancer risk and will provide insight into possible mechanisms through which physical activity might be associated with reduced risk of breast cancer in postmenopausal women.

**Trial registration:**

NCT00359060

## Background

Most of the established risk factors for breast cancer, such as family history of the disease, early age at menarche, late age at menopause, late age at first childbirth or nulliparity are not, or not easily, amenable to intervention. Physical activity is a modifiable lifestyle characteristic that has been associated with breast cancer risk in various studies [1-22] and as such a potential means for the primary prevention of breast cancer. A recent review [23] showed strong evidence for an inverse association between physical activity and postmenopausal breast cancer, with risk reductions ranging from 20–80%. For premenopausal breast cancer, however, the evidence was much weaker. A recently published large cohort study, EPIC, provides additional evidence for a protective effect of physical activity on breast cancer risk [24]. The causal pathway between physical activity and (postmenopausal) breast cancer risk, however, is not clear. Several biological mechanisms that mediate the relation between physical activity and breast cancer are suggested. The most prominent hypothesis is that physical activity affects the hormonal milieu, including sex steroid hormones and metabolic profiles [25-28]. The evidence that estrogens contribute to breast cancer risk is strong and widely accepted. Women with relatively high levels of estrogens have increased risk of developing breast cancer [29-32].

Furthermore, both an early menarche and a late menopause increase risk, probably by increasing lifetime exposure to ovarian hormones [33,34]. Several cross-sectional studies have shown that an elevated level of physical activity is associated with 15–25% lower serum concentrations of estradiol, estrone and androgens in postmenopausal women, even after adjustment for body mass index [35-37]. In addition, a positive energy balance has been associated with (a) increased levels of active estrogens, (b) increased levels of unbound estradiol and (c) increased levels of progesterone [38]. This association can be explained either directly but also indirectly through accumulation of adipose tissue [39,40]. Compared with normal-weight postmenopausal women, obese postmenopausal women have higher blood concentration of both estrone and estradiol and lower concentration of sex hormone binding globulin. In postmenopausal women, increased levels of estrogens due to peripheral conversion (mainly in fat cells) from androgens result from increased body fat, particularly intra-abdominal fat mass. Regular exercise represents an approach to regulate energy balance and to prevent the accumulation of adipose reserves and consequently influence the production of estrogens. Another possible mediator in the relation between physical activity and breast cancer risk may be insulin (resistance). Several studies have shown inverse associations between physical activity and markers for insulin sensitivity independent of body size [41-45].

So far, one study has reported on the effects of an exercise intervention on sex steroid concentration among postmenopausal women. This study observed that a 12-month moderate-intensity exercise intervention in sedentary, overweight women resulted in small but significant decreases in serum estrogens and androgens. These results were restricted to women who lost a certain amount of body fat.

In order to get more insight in the causal pathway between physical activity and breast cancer risk, we designed the *Sex Hormones and Physical Exercise (SHAPE) *study. This study examines the effect of an exercise intervention on sex steroid hormones and insulin in sedentary postmenopausal women and investigates if the amount of total body fat or abdominal fat mediates the effect. If increase in physical activity has a beneficial effect on the sex hormone and metabolic profile of postmenopausal women, it will offer opportunities for breast cancer prevention programs. This paper presents the design and evaluation plan of the SHAPE study.

## Methods/Design

The SHAPE study examines the effects of a 1-year moderate intensity exercise programme on endogenous hormone levels (sex steroid hormones, insulin) associated with breast cancer among sedentary postmenopausal women. The study is designed as a single blind, randomised, controlled trial with two study arms.

We chose to include only postmenopausal women because (a) there is more evidence for an inverse association between physical activity and breast cancer risk in postmenopausal women (b) the incidence of breast cancer is greatest in postmenopausal years (c) the major conversion locus of androgens into estrogens in postmenopausal women is fat tissue – a reduction in fat mass through exercise may be more likely to affect the relative concentrations of estrogens on postmenopausal women than in premenopausal women (d) there are no problems associated with measurements of hormones with timing of the menstrual cycle.

### Study population

The SHAPE study includes 189 healthy postmenopausal women aged 50 tot 69 year, who are sedentary and not currently using postmenopausal hormone replacement therapy. Postmenopausal status is defined as not having menstrual periods for at least 12 months. Being sedentary is defined as less than 2 hours per week of moderate sport activity (e.g., tennis, swimming, running, aerobics, fitness, and volleyball) and not adherent to the international physical activity recommendation. The international physical activity recommendation states that every adult should accumulate 30 minutes or more of at least moderately intense physical activity for at least five days per week [46,47]. The exclusion criteria (see Table 1) are based on factors that might interfere with measurement of endogenous hormones or with the success of the intervention, or that might affect the safety of the participant. We exclude women with a BMI below 22, because these women may have estrogen levels below the detectable levels of a laboratory. The study protocol is approved by the Medical Ethics Committee of University Medical Centre Utrecht before the start of data collection.

**Table 1:** Exclusion criteria of the SHAPE study

< 12 months since last menses
Use of hormone replacement or oral contraceptives in past 6 months
Active life style
Morbidly obese (BMI > 40)
BMI < 22
Currently on or planning to go on a strict diet
Ever diagnosed with breast cancer
Diagnosis of other types of cancer in the past 5 years
Diabetes mellitus or other endocrine related diseases
Disorders or diseases (locomotor, optical, neurological, mental) that might impede the participation in the exercise programme
Alcohol or drug abuse
Maintenance use of corticosteroids
Use of beta blockers
Smoking

### Recruitment

**Figure 1:**
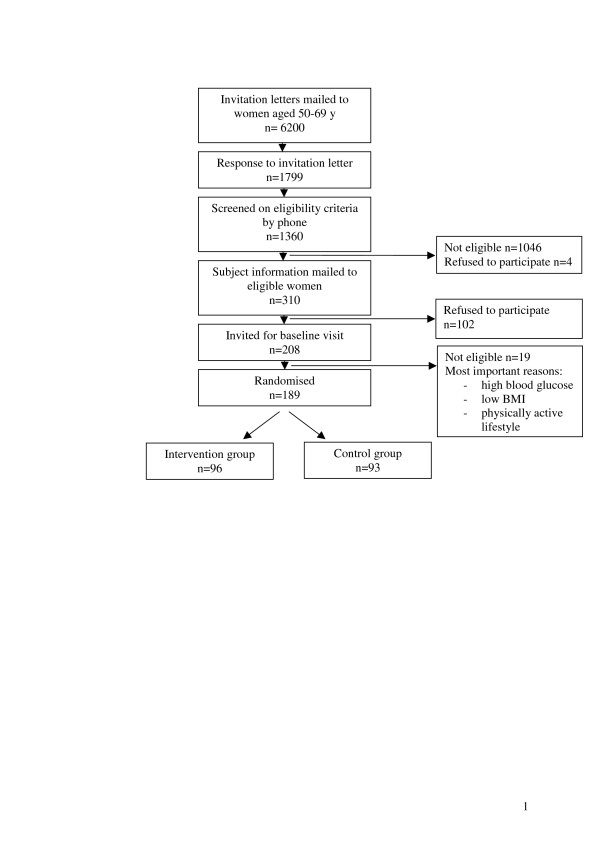
Flow chart of the inclusion of the SHAPE participants.

Participants are recruited through a random selection out of the female inhabitants aging 50–69 years of the following middle sized municipalities in the centre of The Netherlands: Utrecht, Zeist, Bilthoven, Houten, Soest en IJsselstein. Potential candidates receive an invitation letter explaining the goal of the study and a short eligibility questionnaire. Next, the potential candidates are contacted by phone to explain further details and the inclusion criteria list is completed to screen eligibility. Additionally, interested and eligible women receive written study information and are asked to complete and sign an informed consent form. With the women who return their informed consent form an appointment for the baseline visit at our research unit is made. See Figure 1 for the flow chart of the inclusion of the participants.

### Intervention

Eligible women are randomised into an intervention group or a control group. Randomisation is blocked on two categories of waist circumference: < 92 cm and ≥ 92 cm (cut-off level is based on the median value reported in similar women [48]).

Women in the intervention group participate in a combined endurance and strength training programme over a period of 12 months. The programme is organised in a way to optimise fat loss. The goal of the intervention programme is to have participants exercise at least 3 times per week (i.e. twice in a supervised group session and once individual) and to reach a training stimulus in each person. In general, implementation of the exercise goals is done slowly, to reduce chance of injury and to increase participants' adherence and sense of accomplishments.

#### Group Exercise

Twice a week the participants of the intervention group meet for an exercise session of one hour. Each group includes about 15–20 women. A qualified sports instructor facilitates the standardised group sessions according to a protocol. Group exercise takes place in six fitness centres located in the subjects' home town. Classes start with a 10-minute warming up: e.g. exercise to music routines and walking. Then the training is continued with moderate-to-vigorous level of aerobic exercise on 60 – 85% of the age-predicted maximum heart rate. The maximum heart rate is established as 220 minus the person's age in years. The participants receive heart rate monitors to control their training intensity. The training session ends with 25 minutes strength exercises and 5 minutes cooling down. The sport instructor registers the attendance of the subjects. Since it is important that the group exercise sessions are conducted uniformly in all centres, all instructors who participate in the SHAPE study are instructed extensively. In addition, the study coordinator (EM) performs several monitor visits per exercise group to control adherence to the protocol.

Previous experience showed that group exercises are preferred by Dutch women of this age and are better adhered to in the short and long term [49]. However, for budgetary and practical reasons also an individual exercise session is included.

#### Individual Exercise

Once a week the participants are asked to perform an individual home-based exercise session. They receive instructions during the group sessions. The home-based exercise programme consists of 30 minutes brisk walking or cycling with an intensity of moderate-to-vigorous intensity (60–80% of maximal heart rate). Afterwards, the women record the type and duration of their activity in an exercise log, together with the mean heart rate during the training and the BORG-score (6–20) for exertion [50].

### Control group

Participants in the control group are requested to retain their habitual exercise pattern. They also receive newsletters. Although some control subjects may decide to increase their level of physical activity, we believe that it is unlikely that they will sustain in a similar level as the intervention group. Level of physical activity is measured frequently to monitor changes in physical activity (PASE questionnaire) during the course of the intervention.

### Outcomes and measurements

The study participants visit the research unit of the Julius Center at baseline, after 4 months and at the end of the study (See table 2). To evaluate the effects of the exercise intervention and potential confounding factors, data are collected in several ways, i.e., by blood samples, physical examination and questionnaires. Socio-demographic information, reproductive factors, smoking information, past physical activity and medical history is collected by a self-constructed questionnaire at baseline only. Besides blood sampling, anthropometric measurements including dual-energy x-ray absorptiometry (DEXA) and ultrasound of the abdomen, blood pressure measurements and a submaximal exercise test is performed each visit. Also, medication use is assessed each visit. Furthermore, habitual physical activity and diet are assessed at baseline and at the end of the study. Level of physical activity is assessed every four months to investigate changes during the intervention period in both groups either in person or by phone.

**Table 2:** Time schedule of the measurements in the SHAPE study

**Measurement**	**Time (months)**													
	**-0.5**	0	1	2	3	**4**	5	6	7	**8**	9	10	11	**12**

Blood sampling	√					√								√
Anthropometry	√					√								√
Ultrasound abdomen	√					√								√
Whole body DEXA scan	√					√								√
Blood pressure	√					√								√
Submaximal exercise test	√					√								√
Medication use (internet database)	√					√								√
General questionnaire#	√													
Physical activity questionnaire	√													√
Past week activity questionnaire	√					√				√				√
Food frequency questionnaire	√													√
Exercise programme (intervention only)		√	√	√	√	√	√	√	√	√	√	√	√	√
Exercise logs (intervention only)		√	√	√	√	√	√	√	√	√	√	√	√	√

# General questionnaire includes demographic information, reproductive factors, smoking information and medical history

#### Blood samples

Blood samples (30 ml per visit) are drawn between 9.00 and 11.00 AM after an overnight fast in order to determine serum concentrations of estradiol (total, free), estrone, estrone sulfate testosterone, androstenedione, insuline, glucose and sex hormone binding globulin. Blood samples are stored at -70°C. All samples from an individual subject are analysed in the same batch since the batch-to-batch variation can be higher than any woman's likely change in hormones over the year [51]. Serum estrogens, androgens, insulin and sex hormone binding globuline (SHBG) are determined by use of commercially available double-antibody radioimmunoassay kits.

The laboratory "Stichting Huisartsenlaboratorium Oost" in Velp [52] performs all assays.

#### Physical examination

##### Anthropometry

Body weight and height (to the nearest 0.5 kg and 0.5 cm respectively) are measured while the subjects wear light clothes and no shoes using an analogue balance (SECA) and wall-mounted tape measure.

Body fat distribution is measured by the waist- and hip circumference. Waist circumference (to the nearest 0.1 cm) was measured standing at the midway between lower ribs and iliac crest. Hip circumference (to the nearest 0.1 cm) was measured standing over the buttocks. All measurements were taken in duplicate and averaged. Total body fat and body fat percentage are determined by using a whole-body DEXA scan (Lunar, Prodigy™). A whole body scan analyses body composition according to a three-compartment model: fat mass, lean tissue, and bone mineral content. The standard soft tissue analysis is performed using software supplied by the manufacturer. Total body fat is estimated for each subject in kilograms.

Intra-abdominal fat is measured by abdominal ultrasound assessment (Acuson Aspen, Siemens). The ultrasound measure comprises the distance between the peritoneum and the lumbar spine at three predefined places. All measurements are performed longitudinally from one line over the abdomen halfway between the lower rib and iliac crest.

##### Blood pressure

Blood pressure is measured with an automatically tonometer (OMRON M4) after participants have been sitting quietly for at least five minutes.

##### Fitness

Fitness is measured by the maximal oxygen uptake (VO_2 _max). VO_2 _max is determined by a submaximal cycle test: the Fit Test programme on a Life Fitness cycle ergometer. The theory of the test is based on the Ästrand Rhyming Protocol [53], in which, a submaximal heart rate at a known workload is used to predict maximal aerobic capacity. The participants wear light clothing and a heart rate monitor. Prior to each test, the seat height of the cycle ergometer is adjusted for near full extension of the subject's leg while pedalling and is kept the same during the whole study. Before the start of the test, weight, age and gender is entered in the programme. Additionally, a resistance level that suits the subject is chosen. Guidelines for the resistance level have been specified in the manual of the manufacturer. During the test, the subject pedals for 5 minutes using a relatively consistent cadence. The subject should exercise with an intensity of 60 – 85% of the age-predicted maximum heart rate (220 – age). Towards the end of the 5 minutes, the programme measures the heart rate via telemetry. Once a steady heart rate reading is acquired, the programme calculates a VO_2 _max estimate based on the subject's weight, age, gender, selected resistance, and steady-state heart rate. After the test, the subject pedals without resistance for 2 minutes to cool down.

To assess reproducibility of the Fit Test, we tested 25 volunteers twice with an average period of 2.0 ± 1.5 days between the tests. Group means and standard deviations of the first and second VO_2max _measurement were almost identical: 38.9 ± 6.5 ml/kg/min versus 38.8 ± 6.5 ml/kg/min, respectively. The test-retest reliability (i.e. Pearson's correlation coefficient) was 0.993 (P < 0.001). Other reliability indexes (i.e. Intraclass Correlation Coefficient, Standard Error of Measurement and Smallest Detectable Difference) were respectively 0.993, 0.532 ml/kg/min and 1.475 ml/kg/min. This reproducibility study showed that the Fit Test is a reproducible measure of submaximal work capacity.

#### Questionnaires/interviews

A self-constructed questionnaire (general questionnaire) is used to assess level of physical activity, diet, socio-demographic variables, smoking history, medical history and reproductive factors.

##### Habitual-, occupational- and physical activity in the past

The general physical activity questionnaire includes the Voorrips questionnaire measuring habitual activity in elderly subjects [54] and questions concerning occupational physical activity and activity in the past. The Voorrips questionnaire includes household activities, sporting activities and other physically active leisure activities. Altogether it leads to an overall activity score. In the questionnaire, the respondents are asked to report habitual physical activities of the last year. Items on household activities are questions with four to five possible ratings from active to inactive. Sports and other activities are asked as type of activity, hours per week spent on it, and period of the year in which the activity is normally performed. All activities are classified according to work posture and movements. An intensity code, originally based on Bink et al [55], is used to classify each activity.

##### Recent physical activity

The PASE (Physical Activity Scale for the Elderly) questionnaire [56] measures occupational, household and leisure- related activities over a 1-week period. This questionnaire includes 12 categories of physical activity and records the frequency of participation in these activities over the preceeding 7 days. Scoring procedures were derived from motion sensor counts, physical activity diaries and a global activity self-assessment [56]. The PASE generates a single composite score of physical activity that ranges from 0 – 400. We modified the PASE slightly by adding additional answer categories for the duration of the activities. We divided the category "less than 1 hour" into "less than 30 minutes", "30–45 minutes" and "45 minutes – 1 hour" in order to compare the activity level of the participants with the international recommendations for prevention of obesity. The calculation of the PASE score is modified accordingly.

##### Diet

Daily caloric intake, percent daily calories from fat, percent daily calories from carbohydrates and proteins is determined by a food frequency questionnaire [57,58]. The questionnaire consists of 74 items. The questionnaire is completed by the participants at home and subsequently checked by a dietician at the research unit.

##### Medication use

Medication use is asked each visit and registered by use of an internet database. This database is based on the current available medications and uses ATC codes.

### Statistical analysis

All randomised subjects will be analysed according to the intent-to-treat principle. The intention-to-treat principle will assess the intervention effect based on assigned treatment at the time of randomisation regardless of adherence. Demographics and baseline characteristics will be reported descriptively. We will assess whether life style factors that are potentially related to hormone levels might have changed differentially between exercisers and controls, including alcohol use and caloric intake. Differences between the treatment groups in primary and secondary efficacy parameters will be analysed by repeated measurement analyses in SAS and 95% confidence intervals will be calculated. We will also assess effect modification by change in intra-abdominal (and total body fat) and, among exercisers only, by adherence levels.

## Discussion

In this paper, we present the rationale and design of the SHAPE trial which aims to get more insight in the biological mechanism underlying the observed effect between physical activity and breast cancer risk.

The success of the study will depend to a large extent on adherence to the exercise protocol. Factors that enhance adherence to exercise programmes in The Netherlands are group exercise (in stead of individual), a varied programme and a regular instructor [59]. We incorporated all three factors in the SHAPE study as much as possible. First, group exercise comprises the most important part of the total exercise programme. Second, the aerobic component of the exercise programme can be varied to a large extent by the sport instructors. The strength exercises, however, are highly standardised and repetitive which might be less challenging for the women. We choose for this type of strength training for two important reasons: 1) to decrease the risk of injury and 2) since this type of strength training is most optimal to increase muscle mass. Third, we opt for regular instructors for all training groups during the follow-up period, however, in practice there may be some changes during the year. Additional procedures that we use to enhance adherence are: personal feedback, use of exercise logs and newsletters.

It is also of utmost importance that the control group is compliant with the study protocol, e.g., retaining their habitual lifestyle pattern. Women who are randomised to the control group might be disappointed about the assignment and may take up exercise or change their diet. We monitor the level of physical activity of all participants with questionnaires in order to get insight in the actual amount of physical activity and potential cross-over between both treatment arms. Changes in diet are monitored by a food frequency questionnaire.

Six fitness centres and fourteen instructors participate in the SHAPE study. Although the exercise protocol is standardised as much as possible, the attitude and experience of the instructor might influence the actual performance of the participants. In the analysis, we will explore whether outcomes of participants within the same sporting centre are correlated (intracluster correlation). If there is evidence for intracluster correlation, we will account for this in the analysis.

Because of the stringent in and exclusion criteria, the participants of the SHAPE study comprise a selected group of postmenopausal women. This type of preselection will not affect the validity of the study (no selection bias) because randomisation is aimed to ensure comparability of intervention and control group but it may limit the generalisability. For example, since we exclude lean women and women not using hormone replacement therapy, the results might not be generalised to these groups of women.

So far, one study (PATH study) has been published on the effects of exercise interventions on sex steroid concentration in women [60]. The PATH study observed that a 12-month moderate-intensity exercise intervention in sedentary, overweight, postmenopausal women resulted in significant decreases in serum estrogens and androgens. These results were restricted to women who lost body fat, e.g., among women who lost > 2% of body fat, serum estradiol and testosterone concentrations fell by 13.7% and 10.1 % between baseline and 12 months in exercisers compared with a increase of 4.7% and a decrease of 1.6% in controls, respectively [61,62]. Two other controlled physical activity intervention studies in postmenopausal women are under way. These are the ALPHA trial (Alberta Physical Activity and Breast Cancer Prevention Trial) [63] and The NEW Trial (Nutrition and Exercise for Women) [64]. The Alpha trial examines the effect of a 12-month aerobic exercise intervention compared with a usual sedentary lifestyle on several biomarkers of breast cancer risk in 330 sedentary postmenopausal women. The NEW trial is a 4-arm trial examining the effects of diet and exercise induced weight loss on biomarkers of breast cancer risk in 503 sedentary postmenopausal women randomly assigned to dietary weight loss alone, exercise alone, dietary weight loss plus exercise, or usual lifestyle control. Together, these results will shed important light on biological mechanisms by which physical activity influences breast cancer risk. These studies may also give information about the type of exercise needed for breast cancer risk reduction.

In summary, this paper shows the rationale and design of the SHAPE study. The SHAPE study aims to unravel the mechanisms underlying the association physical activity and breast cancer risk. Furthermore, this study evaluates the feasibility of delivering an exercise programme for postmenopausal women.

## Abbreviations

BMI, Body Mass Index; DEXA, Dual-Energy X-ray Absorptiometry; SHBG, Sex Hormone Binding Globulin; VO_2_max, Maximum Oxygen Uptake; ATC, Anatomical Therapeutic Chemical Classification System

## Competing interests

The author(s) declare that they have no competing interests.

## Authors' contributions

EMM, PHMP and AJS designed the study, participated in the coordination of the study and writing of the article. All authors provided comments on the draft and have read the paper.

## Pre-publication history

The pre-publication history for this paper can be accessed here:


